# Expression of Concern: Low-dose chest CT for diagnosing and assessing the extent of lung involvement of SARS-CoV-2 pneumonia using a semi quantitative score

**DOI:** 10.1371/journal.pone.0279045

**Published:** 2022-12-13

**Authors:** The PLOS ONE Editors

This article [1] has been identified as one of a series of submissions for which we have concerns about the reported research ethics approval information and the article’s adherence to PLOS research ethics policies.

PLOS will be investigating these concerns in accordance with COPE guidance and journal policies. Meanwhile, the *PLOS ONE* Editors issue this Expression of Concern.

## References

[1] Leger T, Jacquier A, Barral P-A, Castelli M, Finance J, Lagier J-C, et al. (2020) Low-dose chest CT for diagnosing and assessing the extent of lung involvement of SARS-CoV-2 pneumonia using a semi quantitative score. PLoS ONE 15(11): e0241407. 10.1371/journal.pone.0241407 33141845 PMC7608883

